# A mechanistic alternative to minimal sufficiency as the guiding principle for NCC research

**DOI:** 10.1093/nc/niae014

**Published:** 2024-04-05

**Authors:** Andy Mckilliam

**Affiliations:** Cognition and Philosophy Lab, Monash Centre for Consciousness and Contemplative Studies, Department of Philosophy, Monash University, Room 429, 29 Ancora Imparo Way, Melbourne, VIC 3800, Australia

**Keywords:** neural correlates of consciousness, mechanisms of consciousness, constitutive relevance, mutual manipulability

## Abstract

A central project for the neuroscience of consciousness is to reveal the neural basis of consciousness. For the past 20-odd years, this project has been conceptualized in terms of minimal sufficiency. Recently, a number of authors have suggested that the project is better conceived in mechanistic terms as the search for difference-makers. In this paper, I (i) motivate this mechanistic alternative to minimal sufficiency, (ii) develop it further by clarifying debates about the prospects of leveraging mutual manipulability to distinguish constitutive difference-makers from those that are merely causal, and (iii) explore the implications this has for recent debates concerning the status of the prefrontal cortex. I argue that adopting a mechanistic approach to the neuroscience of consciousness suggests that the prefrontal cortex is part of the neural mechanisms underlying consciousness even if it is not strictly speaking a necessary part.

## Introduction

A central project for the neuroscience of consciousness is to reveal the neural basis of consciousness. For the past 20-odd years, this project has been conceptualized in terms of minimal sufficiency. The objective has been to reveal the neural processes that are minimally sufficient for various states of consciousness (Chalmers 2000, Koch 2004, Koch et al. 2016). In recent years, a number of authors have pointed out problems with this way of conceptualizing the project and a number of modifications and alternative proposals are beginning to emerge (Seth 2009, Neisser 2012, Fink 2016, Miracchi 2017, Klein et al. 2020). One particularly promising alternative appeals to difference-making models of explanation and argues that rather than seeking minimally sufficient conditions, the neuroscience of consciousness is best viewed in mechanistic terms as ‘the search for control variables whose settings have systematic effects on consciousness’ (2020, p. 10).

In this paper, I (i) motivate this mechanistic alternative to minimal sufficiency, (ii) develop it further by showing how mutual manipulability (Craver 2007) can help distinguish constitutive difference-makers from those that are merely causal, and (iii) explore the implications this has for recent debates concerning the status of the prefrontal cortex (Boly et al. 2017, Odegaard et al. 2017). I argue that adopting a mechanistic approach to the neuroscience of consciousness suggests that the prefrontal cortex is part of the neural mechanisms underlying consciousness even if it is not strictly speaking a necessary part.

In The traditional conception of an NCC section, I introduce the traditional concept of a neural correlate of consciousness (NCC) and review some of its shortcomings in the Problems with the traditional NCC concept section. In A mechanistic alternative to minimal sufficiency section, I sketch the difference-making alternative and explain how mutual manipulability—or, rather, matched interlevel experiments—can help distinguish difference-makers that are component parts of the mechanism underlying consciousness from those that are upstream causes. In the Implications for the prefrontal cortex debate section, I argue that adopting this mechanistic approach to the search for the neural basis of consciousness suggests that the prefrontal cortex should be considered a part of the mechanism underlying consciousness even if it is not strictly speaking a necessary part.

## The traditional conception of an NCC

There is considerable variability in how the concept of a NCC is deployed in the literature. My intention in this first section is not to survey all of the various uses of the NCC concept, but rather to focus on what I take to be at its core—the idea that NCCs are to be understood in terms of minimal sufficiency (Chalmers 2000, Koch 2004). As Koch and colleagues write in their recent review: ‘NCCs are defined as the minimum neuronal mechanisms jointly sufficient for any one specific conscious percept’ (Koch et al. 2016, p. 308).

A few points are worth making explicit about this way of conceptualizing NCCs. First, although the aforementioned definition makes explicit reference to conscious perception, NCC research is concerned with more than just states of conscious perception. Researchers are also interested in the neural correlates of conscious thought (Smallwood et al. 2021) and emotions (Tsuchiya and Adolphs 2007), as well as with the neural correlates of global states of consciousness—such as alert wakefulness, drunkenness, or dreaming (Laureys 2005, Siclari et al. 2017). This is usually cashed out by distinguishing two targets for NCC research—content-specific NCCs and state NCCs. Content-specific NCCs are minimally sufficient for local states of consciousness—the visual experience of red and the taste of pineapple. State NCCs, by contrast, are sufficient for a creature being conscious rather than not (Chalmers 2000, Bayne 2007, Hohwy 2009).

Second, the NCC for a given state of consciousness is supposed to be sufficient for that state. Those searching for NCCs are not looking for just any old neural processes that happen to correlate with subjective experience. An NCC for a given state should give rise to that state as a matter of natural necessity.

Third, the NCC for a given state of consciousness is supposed to be the minimal set of neural processes that are required in order for that state of experience to obtain. If mere sufficiency was all that was required, the search for the neural correlates of consciousness would be all too easy—just point to the entire nervous system of an individual in that state—and the job is done. The minimality constraint is intended to capture the screening-off practices of scientists. Activity in the retina may be tightly correlated with conscious vision, but activity in the retina is screened off as an upstream cause. Activity responsible for motor output and language production may be tightly correlated with states of consciousness by virtue of being implicated in the collection of verbal reports, but they are typically screened off as downstream effects. What scientists are doing here, according to the traditional NCC definition, is trying to home in on just those neural processes that are necessary for the conscious state in question—the ‘NCC proper’ as Aru and colleagues call it (Aru et al. 2012; see also Frith et al. 1999, Chalmers 2000, de Graaf et al. 2012).

Before highlighting some problems with conceptualizing the NCC project in this way, it is worth briefly reflecting on the NCC label itself. Given that NCCs are defined in terms of minimal sufficiency, one might wonder why they continue to be called neural ‘correlates’ of consciousness. After all, sufficiency is a much stronger relation than correlation. My watch reading 5 p.m. may correlate with the pub opening, but it is not sufficient for it—I cannot make the pub open early by turning my watch forward I’m sorry to say.

One reason why the term ‘correlate’ is used is that it clearly separates the empirical question about which neural processes are directly related to consciousness from the hotly contested question about the nature of that relationship and of consciousness itself (Hohwy 2007). Correlation is a metaphysically neutral relation. So, identity theorists, realization theorists, and even dualists can all agree that the science of consciousness should proceed by searching for NCCs, even while disagreeing about the metaphysics.

A second reason why the term ‘correlate’ is used is that in the early days—around the turn of the 21st century—there was a heavy focus on contrastive analysis as the primary methodology via which NCCs are to be identified (Baars, 1988, Frith et al. 1999, Chalmers 2000, Hohwy 2007). In a contrastive analysis, researchers leverage binocular rivalry and masking techniques to contrast subjects’ neural activity when they perceive a stimulus consciously with that produced by unconscious perception of the same stimulus (Baars, 1988). A contrastive strategy is also deployed in state-based research by contrasting neural activity associated with various stages of anaesthesia (Alkire and Miller, 2005) and by contrasting dreaming with dreamless sleep (Siclari et al. 2017). The contrastive method, it is often suggested, provides only correlational evidence and so is only capable of revealing neural correlates of consciousness.

How compelling are these motivations today? In my view, not very. First, while metaphysical neutrality is a virtue in this context, correlation is not the only relation that is metaphysically neutral. As Miracchi (2017) and Klein et al. (2020) point out, the difference-making relation can also be viewed as neutral with respect to the relevant metaphysical issues. To say that one variable makes a difference to another is just to say that one can manipulate the latter by intervening to change the former. Identity theorists, realization theorists, and even dualists can all agree that one can manipulate states of consciousness by intervening into the brain, even while disagreeing about the metaphysics.

How about methodological considerations? The case here is not strong either. First, it is debatable whether the evidence from contrastive neuroimaging studies really is merely correlational. As I will explain in more detail in A mechanistic alternative to minimal sufficiency section, contrastive neuroimaging studies can be interpreted through an interventionist’s lens as involving top-down interventions—experiments in which researchers elicit changes in the phenomenon of interest and detect changes in the underlying mechanisms—suggesting that they are capable of revealing more than mere correlation (Craver 2007). Second, even if contrastive analysis does only provide correlational evidence, the conclusions researchers seek to draw from them typically concern difference-makers not mere correlates (Klein 2017). Third, in recent years, there has been an increased effort to integrate evidence from contrastive studies with more obviously causal evidence from studies involving lesions and direct neural stimulation (Aru et al. 2012, de Graaf et al. 2012, Koch et al. 2016, Raccah et al. 2021, Michel 2022).

It seems then that neither metaphysical nor methodological considerations speak in favour of continuing to refer to the project as the search for neural correlates of consciousness. That said, the term does have a rather nice ring to it, and it is already deeply entrenched in both the scientific and popular literature. Displacing it would not be easy. In any case, I will not attempt to do so here, since my quarrel is not with the NCC label itself, but rather with conceiving of the objectives of the neuroscience of consciousness in terms of minimal sufficiency.

## Problems with the traditional NCC concept

In recent years, a number of researchers have pointed out that there is a tension between how NCCs are defined and what researchers are actually searching for. Consider, for example, research into the neural basis of conscious face perception. Neuroimaging studies involving binocular rivalry have shown that experiences of faces are systematically correlated with increased activity in the fusiform face area (FFA) (Tong et al. 1998). Lesion studies have shown that localized damage to the FFA can selectively impair a subject’s ability to experience faces while sparing many other perceptual faculties (Corrow et al. 2016). Evidence from direct brain stimulation during epilepsy screenings reveals that it is possible to selectively manipulate subjects’ experiences of faces by stimulating neurons within the FFA (Rangarajan et al. 2014). Together, these findings suggest that the FFA plays a particularly central role in the neural mechanisms responsible for conscious experiences of faces and is ‘close to qualifying as a content-specific NCC’ for face experience (Koch et al. 2016, p. 315).

These results are undoubtedly important, but the FFA does not really live up to the definition of an NCC as being sufficient for a conscious experience of a face. As Block and Kanwisher have argued, no one thinks that an FFA excised from the brain and kept active in a Petri dish would be capable of generating conscious experiences of faces (Kanwisher 2001, Block 2005). Indeed, it is natural to think that the FFA is only even relevant to conscious experiences of faces when appropriately connected to the rest of a conscious organism.

Chalmers (2000) and Block (2005) have attempted to clarify the picture by appealing to the distinction between ‘core’ and ‘total’ realizers of a functional state (Shoemaker 2007). Block writes:

The total NCC of a conscious state is—all by itself—sufficient for the state. The core NCC is the part of the total NCC that distinguishes one conscious state from another—the rest of the total NCC being considered as the enabling conditions for that conscious experience. (Block 2005, p. 47)

This is helpful. It clarifies that content-specific NCCs are not sufficient for the conscious state in question all by themselves; they are only sufficient in context. But the core/total distinction has problems of its own. In relegating everything outside the core as mere enabling conditions, the core/total distinction (i) gives the impression that neural regions that code for specific content are the only theoretically interesting findings and (ii) occludes from view the central question in the neuroscience of consciousness today: what, in addition to activation of content-specific NCCs, is required for conscious perception.

Consider again the relationship between the FFA and conscious experiences of faces. The issue here is not just that a range of enabling conditions—such as a steady supply of oxygen and glucose and appropriate sensory inputs—are required for the FFA to function correctly. The issue is that the FFA is merely a representational system, and the brain can represent information both consciously and unconsciously. The FFA is only one part, albeit an important part, of the neural mechanisms required for a subject to consciously experience a face. In order to give rise to a conscious experience of a face, activity within the FFA needs to be coupled with what has variably been called the ‘non-differentiating NCC’ (Hohwy and Bayne 2015), the ‘general neural correlate of (perceptual) consciousness’ (Marvan and Polák 2020), and the ‘difference-makers that transform unconscious contents into conscious contents’ (Michel and Morales 2020).

Exactly what is required to transform an unconscious neural representation into a conscious one is an open empirical question and a vibrant area of research on which the field is currently divided. Some authors argue that it is only when neural representations are integrated with mechanisms in and around the prefrontal cortex that they become conscious (Odegaard et al. 2017, Michel 2022). Others argue that integration into the prefrontal cortex is only required for making contents available for report and high-level cognition, and what renders them conscious is integration with other neurally encoded sensory content in the back of the head (Lamme 2006, Boly et al. 2017).

What we see here is that research into the mechanisms underlying conscious perception can be seen as engaging in two distinct projects. One project is to identify neural systems that code for specific contents. This project is not in fact interested in neural mechanisms that suffice for consciousness. Rather, it is interested in the mechanisms that, as Koch and colleagues put it, ‘determine a particular phenomenal distinction within an experience’ (Koch et al. 2016, p. 308). The other project is investigating which neural mechanisms collude with areas that code for specific content in the generation of a conscious percept. This project is not really after neural mechanisms that suffice for consciousness either. Those who argue that mobilization into the prefrontal cortex is necessary for conscious perception do not, as far as I am aware, think that activity in the prefrontal cortex suffices for consciousness all by itself. Rather, they view it as the difference-maker that transforms unconscious contents into conscious contents (Michel and Morales 2020, p. 1).

One might suppose that when these two projects are combined, they yield neural mechanisms that jointly suffice for consciousness. But even this is debatable. Here, we run into a methodological issue that John Searle identified early on. It is not clear that we can answer questions about what mechanisms are truly sufficient for consciousness solely by investigating the mechanisms underlying conscious perception. After all, manipulating what a creature is conscious of is not the same thing as manipulating whether that creature is conscious (Searle 2000, Bayne 2007, Hohwy 2009).

To summarize then, describing these projects investigating the neural basis of conscious perception as revealing neural states that are minimally sufficient for consciousness is at best awkward and misleading. At worst, it is patently false.

In addition to being descriptively awkward, by emphasizing neural states that code for specific contents as being the most theoretically interesting findings, the traditional conception of an NCC is also leading researchers astray. For example, in their 2016 review, Koch and colleagues suggest that the objective of research exploring the neural basis of global states of consciousness is to reveal ‘the neural substrates supporting conscious experiences in their entirety, irrespective of their specific contents’, which they take to be ‘the union of the sets of content-specific NCC for all possible contents of experience’ (2016, p 308).

There are two issues with this. First, if content-specific NCCs are not by themselves sufficient for consciousness, why think that simply bundling them together into a set would make the relevant difference? Advocates of cognitive theories of consciousness will object. As they see it, neurally encoded content needs to be rendered globally available for cognition in order to become conscious, and this is thought to involve regions that do not code for specific content. Advocates of recurrent processing theory (Lamme 2006) and the integrated information theory (Tononi et al. 2016) should also object, for, according to these views, it is only when the information encoded in content-specific NCCs is appropriately integrated that they give rise to consciousness.

Second, state-based NCC research arguably needs to do more than merely reveal the neural substrate that supports conscious experience. In addition to feeling like something from a subjective perspective, conscious processing also imbues subjects with a suite of capacities for engaging with the world and with their own mind. In recent years, several authors have argued that global states of consciousness are best characterized, at least in part, in terms of these consciousness-related capacities (Bayne, Hohwy, & Owen 2016; Mckilliam 2020).

Compare the conscious life of someone who has the capacity to endogenously direct their attention with that of someone who lacks that capacity. There is a very real sense in which the conscious experiences of the former are ‘part of [their] own making’ (Watzl 2017, p. 44). By actively directing their attention to the sound of the wind through the leaves, or the feeling of grass under their feet, or the subtle blend of flavours in a cup of coffee, they can bring those contents to the centre of their conscious field and thereby ensure that they enjoy high-quality representations of those conscious contents. Similarly, by moving their body, they can deliberately alter the sensory input they receive and, by virtue of doing so, play an active role in the production of their future experiences. In disordered states of consciousness such as akinetic mutism, the vegetative state, and the minimally conscious state, it is not just the quality of occurring experiences that are impoverished. These subjects lack or have greatly diminished capacities to orchestrate the unfolding of their conscious life.

Given that part of the motivation for NCC research is the hope that it will allow us to design new therapies for patients in disordered states of consciousness (Boly et al. 2017), NCC research should investigate the full suite of consciousness-related capacities, not merely those that are minimally sufficient for subjective experience. If a therapy restored to a patient the capacity for rich perceptual experiences but left them incapable of planning, reasoning, hoping, desiring, and generally engaging in conscious life, it is far from clear that it would be worth pursuing.

This has further methodological implications. To date, within the state, no report paradigm has been championed as ‘the cleanest way to identify the Full NCC’ (Boly et al. 2017, p. 9607). In a within-state no-report paradigm, researchers take advantages of natural fluctuations in consciousness that takes place within a single global state in order to control for variations in behavioural capacities across distinct global states. For example, Siclari and colleagues contrasted neural activity of dreaming and dreamless sleep and found that dreaming involved increased activity primarily in the posterior hot zone (Siclari et al. 2017). But if global states of consciousness are best characterised in terms of consciousness-related capacities, then this methodology is inappropriate precisely because it treats consciousness-related capacities as confounds to be controlled for.

To summarize, conceptualizing the objectives of the neuroscience of consciousness as revealing NCCs is (i) descriptively awkward because content-specific NCCs are not sufficient for consciousness, (ii) occludes from view the question of what transforms unconscious content into conscious content, and (iii) has led researchers to mistakenly treat the suite of capacities that conscious processing imbues us with as mere confounds to be controlled for. Admittedly, until recently, there has been no clear out alternative to the NCC framework and in the absence of an alternative, it is understandable that researchers have stuck with the NCC concept despite its flaws. In the next section I set about developing an alternative.

## A mechanistic alternative to minimal sufficiency

In a recent paper, Klein, Hohwy, and Bayne argue that the neuroscience of consciousness is best viewed in difference-making terms as ‘the search for control variables whose settings have systematic effects on consciousness’ (Klein et al. 2020). To say that X is a difference-maker with respect to Y is just to say that one could, at least in principle, manipulate Y by intervening to change X even while all other influences on Y are held fixed (Woodward 2003).

One advantage of the difference-making framework is that the strength of difference-making relationships varies along a range of dimensions in a way that minimal sufficiency does not. One of these dimensions concerns specificity (Woodward 2010). Some difference-makers have specific effects, while others are more generic. For example, the electrical mains is a difference-maker with respect to the sounds emanating from the radio, but it is not a very specific one. Cutting the power to the mains will turn the music off, but it will also affect the television, the fridge, the lights, and so on. The radio’s on/off switch provides a much more specific locus of intervention. Another dimension of variability is systematicity. While the on/off switch is highly specific, it only allows us to toggle between two states—sound on and sound off. The volume knob, by contrast, provides fine-grained control of the volume. Importantly, not only do many states of the knob map to many states of volume, they do so in a systematic way. Volume increases in a linear fashion with clockwise rotation. This systematicity is important because it allows us to make predictions beyond the cases we have observed—a point Chalmers rightly emphasized early on (Chalmers 2000, p. 23).

A second advantage of the difference-making framework is that unlike minimal sufficiency, difference-making has no difficulty in accommodating the fact that many of the mappings that the neuroscience of consciousness is interested in are highly context sensitive. Outside of the realm of physics, difference-making relationships are rarely universal. Rather, they hold only given some range of background conditions. The radio’s volume knob, for example, only offers a means of manipulating the sounds emanating from a radio when the radio is turned on and connected to the mains.

A third advantage is that difference-making is well suited to accommodate interaction effects between difference-makers. For example, the radio’s on/off switch does not just influence whether there is sound but also influences whether the difference-making relationship between the volume and the volume knob is online. Turning the volume knob only makes a difference to the volume when the radio is switched on. When it comes to modelling the causal structure of a system, these ‘second-order invariants’ are particularly important (Klein and Barron 2020).

Together, this allows for a more nuanced treatment of numerous debates in consciousness science. Consider the debate over the status of V1. On the one hand, subjects with V1 damage report not being conscious of visual stimuli in their blind hemifield, even though information about the stimuli is registered in their brain (Weiskrantz 1996). It has been shown that visual experience can be disrupted by intervening to inhibit feedback back to V1 from higher sensory areas (Pascual-Leone and Walsh 2001). On the other hand, V1 does not appear to code for specific content—the activity of most V1 neurons is correlated with the stimulus and not with the conscious percept (Blake and Logothetis 2002). Given these data, it is unclear whether V1 should be included within the minimally sufficient conditions for conscious vision. Some say yes (Block 2005, Lamme 2006), and others say no (Koch et al. 2016).

Adopting the difference-making framework allows us to clarify the picture here. While content-specific areas like the FFA are systematic difference-makers with respect to particular aspects of conscious experience, V1 is more switch-like. Rather than coding for particular content, it influences whether content-specific difference-makers within the visual domain are online.

At this point, one might object that there are many things that make a difference to consciousness, and the neuroscience of consciousness is primarily interested in only a relatively small subset of these. To put the point bluntly, some properties of perceptual stimuli external to the head are systematic difference-makers with respect to consciousness too. One can systematically manipulate experiences of colour by changing the hue of the percept. But these are treated as upstream causes of conscious states. And the science of consciousness is, it is generally supposed, interested in finding the neural processes that constitute, or realize, or are the supervenience base for consciousness. In other words, how are we to understand the screening-off practices in science if not as the search for minimal sufficiency?

One response is to object to this interpretation of the screening-off practices in science. According to this view, the reason researchers engage in screening-off practices is to identify difference-makers that allow for the most predictive and explanatory purchase. Klein, Hohwy, and Bayne adopt this view. They argue that ‘the mere fact that something is external to the head does not mean that it is not a useful [difference-maker] for building a science of consciousness’, and they support this view by pointing out that ‘consciousness science can and does benefit from the sort of careful, systematic study of stimuli done by perceptual psychophysics’ (2020, p15).

Here, I want to develop an alternative response to this question, one that does not give up on the idea that there is theoretical utility in distinguishing causes from constituents. The alternative is to turn to the strategies deployed in the search for mechanisms elsewhere in science for guidance. This brings to light a fourth advantage of the mechanistic alternative to minimal sufficiency—it comports well with the new mechanistic philosophy of science according to which the sciences most relevant to the mind—biology, neuroscience, and psychology—are structured around the development of multilevel mechanistic models that describe both intralevel and cross-level difference-making relationships. The search for mechanisms is not just the search for difference-makers and contrastive explanations but also involves identifying the entities, activities, and organizational principles that explain how things work (Machamer et al. 2000, Bechtel and Abrahamsen 2005, Craver 2007). The advantage of mechanistic knowledge is that when we know how things work, we are in a position to fix them when they break and manipulate them so that they work for us (Craver and Darden 2013).

Before proceeding, a note on terminology is in order. Initially, mechanistic philosophers of science primarily focused on describing the search for mechanisms in science without worrying too much about the metaphysics (Machamer et al. 2000, Bechtel and Richardson 2010, Craver and Darden 2013). More recently, metaphysically minded philosophers have begun to think carefully about the metaphysical foundations of the mechanistic philosophy of science (Baumgartner and Gebharter 2016, Krickel 2018). Although both the descriptive and the metaphysical projects deploy the term ‘constitutive relevance’, it is not clear that they do so in precisely the same way. Those engaged in the descriptive project tend to think that we ‘philosophers should be guided in [our] thinking about constitutive relevance by attending to the interlevel experiments scientists use to test whether something is a component in a mechanism’ (Craver et al. 2021, p. 8809). As a result, they treat ‘constitutive relevance’ as synonymous with ‘component in a mechanism underlying a phenomenon’. By contrast, at least some authors engaged in the metaphysical project appear to have a more restrictive conception of ‘constitutive relevance’ in mind, according to which only a subset of the components in a mechanism—e.g. the nonredundant components—are truly constitutive of the phenomenon (Couch 2011, Baumgartner and Gebharter 2016). Here, I adopt the former, more liberal conception of constitutive relevance, but in doing so, I intend no strong claims about the semantics of the term ‘constitution’. Those who prefer a more restrictive conception of ‘constitutive relevance’ can read what follows as having implications only for whether some variable be included in a model of the mechanism underlying a phenomenon.

### Mutual manipulability: a defeasible guide to constitutive relevance

In the search for mechanisms, scientists deploy a combination of top-down and bottom-up interventionist experiments (Craver 2007). In a top-down experiment, scientists set up conditions that elicit the phenomenon of interest and detect the behaviour of some hypothesized component. In a bottom-up experiment, they inhibit or excite a component and then detect how that changes the way the phenomenon of interest is manifest. For example, research into the neural mechanisms underlying spatial memory benefitted from both top-down experiments—experiments that placed rats in familiar mazes that prompted the deployment of spatial memory and detected activity in the rats’ hippocampus as they navigated the maze (O’Keefe and Dostrovsky 1971)—and bottom-up experiments—experiments that deployed strategies to inhibit the activity of the rats’ hippocampus and subsequently detected the effect this had on their ability to navigate familiar mazes (Morris et al. 1982).

When the results of top-down and bottom-up experiments match—i.e. when activating the phenomenon causes the component to behave in a particular way and preventing that component from behaving in that way yields detectable changes in how the phenomenon is manifest—scientists typically infer that the component in question is part of the mechanism for that phenomenon (Craver 2007, Craver and Darden 2013, Craver et al. 2021).

What licences this inference? Craver has argued that the guiding principle here is mutual manipulability. It is, in effect, an extension of the principle underlying inferences about causation. If one variable is causally relevant to another, then it should be possible to manipulate the latter by intervening to change the former even while all other influences are held constant (Woodward 2003). Craver suggests that in cases of constitutive relevance, manipulability will often go both ways and suggests that mutual manipulability provides a sufficient condition for inferences about constitutive relevance. In other words, Craver argues that if (i) it is possible to manipulate the phenomenon via a surgical intervention that changes the behaviour of a component and (ii) it is possible to manipulate the behaviour of the component via a surgical intervention that elicits or augments the phenomenon, then the component is constitutively relevant to—it is part of the mechanism underlying—that phenomenon (Craver 2007, pp. 152–160).

However, extending the interventionist toolkit to accommodate constitutive relationships has proven to be far from straightforward, and there is now a large and thorny literature debating the viability of mutual manipulability, sketching amendments, and offering alternatives (Couch 2011, Harbecke 2010, Woodward 2014; Baumgartner and Casini 2017, Krickel 2018, Prychitko 2019, Craver et al. 2021).

The central concern in this literature has been that interlevel experiments either (i) imply interlevel causal relations or (ii) violate the norms of Woodwardian interventions and so cannot provide an appropriate analysis of constitutive relevance (Baumgartner and Gebharter 2016; see Kästner and Andersen 2018 for an excellent review, Craver et al. 2021 for a reply). Here, I will set these complications aside because even if mutual manipulability does not provide necessary and sufficient conditions for constitutive relevance—indeed, even if it cannot in principle do so—mutual manipulability can still provide a defeasible guide to constitutive relevance. We just need to be aware of its limitations.

First, as Craver himself pointed out, mutual manipulability is not a necessary condition for mechanistic parthood. Passive components may not meet this condition (Craver 2007, p. 159; see also Craver et al. 2021, p. 8824). Consider the myelin sheath. The job of the myelin sheath is to insulate the axon, thereby greatly increasing the speed at which an action potential can travel down it. The myelin sheath arguably does not satisfy the mutual manipulability criterion. While it is possible to manipulate the speed of an action potential by removing the myelin sheath, triggering an action potential does not affect the myelin sheath’s capacity to insulate the axon in any detectable way. Nonetheless, the myelin sheath plausibly is a constitutive part of myelinated neurons. It is neither a cause nor an effect of the firing of an action potential. Nor does it seem to be a background condition that enables neurons to fire.

Second, without the additional requirement that the lower-level variable in question be a spatiotemporal component of the whole, mutual manipulability may not be sufficient for determining constitutive relevance either (Leuridan 2012, Klein 2018). For example, it is possible to manipulate the amount of oxygen in a room by lighting a candle. It is possible to manipulate a candle flame by intervening to change the amount of oxygen it has access to. But in most cases, the presence of oxygen in the environment would be treated as a background condition for, rather than as partly constitutive of, the flame’s burning.

Of most relevance to debates in consciousness science, however, is the fact that mutual manipulability only tells us what to do when our interlevel experiments involve ideal interventions. It does not tell us when our interlevel experiments are confounded. For example, having a rat navigating a maze will also engage its motor cortex, and lesioning its motor cortex will inhibit its ability to navigate the maze. But it would be too quick to infer that the motor cortex is constitutively relevant to spatial memory. The motor cortex may be on the causal path between the phenomenon of interest—spatial memory—and our method for detecting it—navigation behaviour. This is particularly relevant to consciousness science since one of the central challenges in consciousness science is to disentangle neural processes constitutively relevant to consciousness from those required to elicit introspective reports (Block 2007, Tsuchiya et al. 2015, Phillips 2018). Mutual manipulability provides no silver bullet for resolving this challenge; indeed, it presuppose a solution. To determine when interlevel interventions are confounded in this way, researchers must appeal to other considerations, such as the specificity and systematicity of the effects, to identify interlevel experiments that manipulate upstream causes and downstream effects.

Despite these limitations, mutual manipulability can still provide a useful guide to constitutive relevance in consciousness science. In the remainder of this section, I will argue that not only can researchers appeal to mutual manipulability to distinguish causal variables from constitutive variables, in fact they already do.

### Mutual manipulability in consciousness science

The search for mechanisms in consciousness science can be understood as deploying a combination of top-down and bottom-up experiments to identify neural systems, activities, and organizational features that are constitutively relevant to consciousness.

Consider, for example, the methodology deployed by Rangarajan and colleagues in their study involving the direct stimulation of face-selective areas in epilepsy patients (2014). Subjects were ‘directed to attend to a specific person, face, or object, or sensation’, then an electric current was applied to a predetermined region of cortex, and subjects were asked to ‘recount any perceptual effects or changes they experienced’ (Rangarajan et al. 2014, p. 12830). They found that by applying current directly to certain regions within a patients fusiform gyrus, they could selectively manipulate their ongoing experience of faces while leaving other aspects of consciousness unchanged (2014). This is a bottom-up experiment in which they intervened to manipulate the behaviour of a component in the underlying mechanism and detected changes in the higher-level phenomenon.

Lesion studies can be understood as experiments involving bottom-up interventions too. In lesion studies, researchers deploy neuroimaging techniques to determine that some region of cortex has been damaged and investigate the effects this has on the subject’s consciousness-related capacities. True, in this case, is that the intervention is not deliberately administered by the researcher, and psychological deficits will often provide the initial clue that a lesion is present. But this does not prevent us from understanding lesion studies as experiments involving bottom-up interventions in which some component in the underlying mechanism has been changed and the effects on the phenomenon of interest are detected.

By contrast, neuroimaging studies can be understood as top-down interventionist experiments (Craver 2007, Craver and Darden 2013). In these experiments, researchers put subjects into conditions that elicit a particular conscious state and detect changes in the underlying mechanism. The reason that binocular rivalry and masking techniques have played such a central role in consciousness science is that they provide more surgical top-down interventions. They allow researchers to elicit certain conscious states while controlling for perceptual processing.

Moreover, it is when the evidence from top-down neuroimaging experiments converges with the evidence from bottom-up experiments involving lesions and direct brain stimulation that scientists are particularly confident in saying that that the region of interest is close to qualifying as an NCC (Koch et al. 2016, p. 315). We can infer that perceptual stimuli—e.g. pictures of faces—are merely causally relevant to face experiences, at least in part because one cannot manipulate perceptual stimuli by intervening to change perceptual experiences of those pictures. This suggests that not only can consciousness scientists appeal to mutual manipulability to identify neural processes that are constitutively relevant to consciousness, in fact they already do (Aru et al. 2012, p. 742, Miller 2014).

However, it is important not to overstate the utility of mutual manipulability as a tool for consciousness science. First, as mentioned earlier, by itself, mutual manipulability provides no guidance for establishing when interlevel interventions are confounded. But also, mutual manipulability is a relatively blunt instrument; too blunt to draw the distinctions that consciousness researchers want to be able to draw. Mutual manipulability may be useful for distinguishing constitutively relevant variables from causes and effects, but it will not help identify particularly salient components. For that purpose, mutual manipulability needs to be scaffolded with details about the systematicity, specificity, and robustness of the difference-making relationships emphasized by Klein et al. (2020).

To summarize, according to the mechanistic alternative to minimal sufficiency, rather than seeking neural processes that are minimally sufficient for various states of consciousness, the neuroscience of consciousness should be (and arguably is) seeking variables that stand in various difference-making relationships to consciousness. It can (and arguably does) appeal to mutual manipulability as a defeasible criterion for distinguishing causal difference-makers from those that are constituent parts of the mechanisms underlying consciousness. It should seek to integrate these constitutively relevant neural processes into a multilevel model of the mechanisms underlying consciousness. Admittedly, there is much work to be done articulating the details of this mechanistic alternative. In the remainder of the paper, I want to apply the framework to one of the central debates in consciousness science—whether the prefrontal cortex should be treated as a part of the neural mechanism underlying consciousness.

## Implications for the prefrontal cortex debate

A central debate in the neuroscience of consciousness in recent years has concerned the status of the prefrontal cortex. All parties agree that the prefrontal cortex is crucial for intelligent behaviour and cognitive control. They also agree that the prefrontal cortex plays at least an important causal role in modulating ongoing conscious experience. What they disagree on is whether the prefrontal cortex should be considered a constitutive part of the neural basis of consciousness.

Minimal sufficiency appears to be the principle guiding this debate. The debate has been over whether prefrontal involvement is necessary for consciousness (Boly et al. 2017, Odegaard et al. 2017, Raccah et al. 2021, Michel 2022). If it is not necessary, then it is not part of the minimally sufficient conditions for consciousness, and therefore, it is not part of the neural basis of consciousness. Here, I want to develop the case that, from a mechanistic perspective, the prefrontal cortex should be treated as a part of the neural mechanisms underlying consciousness even if it is not a necessary part.

To see why this might be the case consider an automotive example. Suppose we want to identify the parts of my car that play a constitutive role in making the wheels spin. We can appeal to matched interlevel experiments to screen off the starter motor as an upstream cause. While it is possible to manipulate the mechanism that makes the wheels spin by intervening into the starter motor, once the engine is up and runs the starter, motor’s job is done. We cannot manipulate the starter motor by surgically intervening to change the behaviour of the mechanism. For example, pressing your foot on the accelerator to rev the engine has no effect on the starter motor. Similarly, we might screen off the tachometer as causally downstream of the mechanism that makes the wheels spin, at least partly on the grounds that intervening into the tachometer has no effect on the behaviour of the mechanism. But what should we make of the turbocharger?

A turbocharger is essentially an air compressor that is powered by the exhaust and feeds compressed air back to the engine. By supplying the engine with compressed air, the addition of a turbocharger increases both the power output and the efficiency of the engine. On the one hand, we know that a turbocharger is not a necessary component for a car to have. Cars without them function just fine. Even in cars that do have them, they are typically only operative at high revs. On the other hand, in cars that do have turbocharges, there is reason to think that they are constitutively relevant components.

One reason for thinking that turbos are constitutively relevant to the car’s mechanism appeals to mutual manipulability. Turbos meet the criterion for mutual manipulability. It is possible to manipulate the activity of the turbo via a top-down intervention that changes the behaviour of the mechanism as a whole—e.g. pressing your foot on the accelerator. It is possible to manipulate how the car runs via a bottom-up intervention that inhibits the activity of the turbo. Inhibiting the turbo will influence both the efficiency and the power output of the car.

Another reason for thinking turbos are constitutively relevant appeals to the elimination of alternatives. Given the intimate feedback between the engine and the turbo—the activity of the engine causally affects the turbo, which causally affects the engine—it would be odd to lump the turbo together with either the starter motor as an upstream cause or the tachometer as a downstream effect. Perhaps one might suppose that the turbo is an enabling condition. But if so, it is a very different kind of enabling condition than, say, the presence of oxygen in the atmosphere. After all, the oxygen in the atmosphere is not a spatiotemporal part of the car, the turbo is.

A third reason for treating turbos as constitutively relevant is pragmatic. Part of the motivation for the search for mechanisms is that mechanistic models allow us to predict how a system will behave in counterfactual situations. Including the turbo in a model of the car’s mechanism allows for more accurate predictions about how it will respond when we press on the accelerator. This suggests that even though turbos are not essential features of cars, in cars that do have them, they are best treated as components of the mechanism via which they operate. This is true even when the car is idling, and the turbo is not actively contributing to making the wheels’ spin.

Something analogous, I suggest, may hold for the prefrontal cortex. Even if Boly and colleagues are right and consciousness can occur in the absence of prefrontal cortex involvement, given the intimate causal feedback between prefrontal cortex and sensory cortex in neurotypical subjects, in those creatures lucky enough to possess an intact prefrontal cortex, the prefrontal cortex is best treated as constitutively relevant to the mechanisms underlying consciousness.

The case for this is clearest when we consider research into the mechanisms underlying global states of consciousness. As mentioned earlier, global states of consciousness are best conceptualized partly in terms of consciousness-related capacities. Even if bilateral lesions to prefrontal cortex do not obliterate the capacity for conscious experience entirely, they clearly impact the agent’s ability to engage in conscious life.

With respect to global states of consciousness, the prefrontal cortex satisfies the conditions of mutual manipulability. It is possible to manipulate global state via bottom-up interventions that inhibit or stimulate the prefrontal cortex. Lesions to prefrontal cortex often leave patients incapable of engaging in goal-directed actions (Boly et al. 2017, Odegaard et al. 2017). It is also possible to rouse rats from anaesthesia via a cholinergic stimulation of their prefrontal cortex (Pal et al. 2018). It is possible to manipulate the behaviour of the prefrontal cortex via a top-down intervention that manipulates global state. The induction of the psychedelic state via the administration of Psilocybin produces detectable changes in prefrontal activity (Carhart-Harris et al. 2012), so too does sedating someone with anaesthetics (Bonhomme et al. 2019) and becoming lucid while dreaming (Dresler et al. 2012). This suggests that even if the involvement of the prefrontal cortex is not necessary for subjective experience, it should be treated as constitutively relevant to the mechanisms underlying global states of consciousness.

## Conclusion

In this paper, I have developed and motivated a mechanistic alternative to minimal sufficiency. According to this alternative, the science of consciousness should aim to uncover neural components, activities, and organizational properties that make a constitutive difference to consciousness and integrate them into multilevel mechanistic models that capture how these various difference-making relationships interact. I have argued that matched interlevel experiments not only can help distinguish constituents from causes but also emphasized the limitations of that strategy. Finally, I have considered the implications this mechanistic alternative has for debates over the status of the prefrontal cortex. I have suggested that from a mechanistic perspective, the prefrontal cortex ought to be considered part of the neural mechanisms underlying consciousness even if it is not strictly speaking a necessary part.

## Data Availability

No new data were generated or analysed in support of this research.
